# The disparity between funding for eye research vs. the high cost of sight-loss in the UK

**DOI:** 10.1038/s41433-022-02228-7

**Published:** 2022-09-27

**Authors:** Jennifer M. Dewing, Andrew J. Lotery, J. Arjuna Ratnayaka

**Affiliations:** 1 Clinical and Experimental Sciences, Faculty of Medicine, University of Southampton, MP806, Tremona Road, Southampton, SO16 6YD UK; 2 Eye Unit, University Hospital Southampton NHS Foundation Trust, Southampton, SO16 6YD UK

**Keywords:** Scientific community, Medical research

One in five individuals are estimated to experience significant sight-loss during their lifetime [1]. In the UK, ~2.5 million individuals suffer from some degree of visual impairment, with an additional 350,000 people registered as partially sighted or blind [1]. These figures are expected to show a 40% increase by 2050 [2]. Common eye conditions and their estimated numbers in the UK are: age-related macular degeneration (AMD: 700,000), diabetic retinopathy (144,000), inherited eye disorders (100,000), glaucoma (500,000) and cataracts (500,000) [2]. Although cataracts can be treated surgically, with ~400,000 procedures carried out annually, some eye conditions can only be managed palliatively, whilst others have no treatment whatsoever. AMD is the most common form of irreversible sight-loss amongst UK adults. In addition to the 700,000 AMD patients mentioned above, there are ~1.2 million people living with early stages of the disease [2]. AMD causes the loss of central vision, affecting an individual’s ability to read, write, drive or recognise faces. Early AMD is reported in 9.8% of those aged ≥65 years [3], with 12.2% of individuals over 80 suffering from late stages of the disease [4]. A significant proportion of ophthalmic procedures in the National Health Service (NHS) are related to the treatment of AMD, specifically the administration of intravitreal anti-vascular endothelial growth factor (VEGF) injections every 4–8 weeks for managing the neovascular/end-stage form of the disease. The remaining patients, accounting for ~50% of late-stage AMD cases, present with geographic atrophy [5], which has no meaningful treatment. An example of inherited retinal diseases (IRDs) is Sorsby fundus dystrophy, which is clinically indistinguishable from AMD without genetic testing, and is managed only through regular anti-VEGF injections [6]. Despite being individually rare, collectively, IRDs are relatively common, and is the most common cause of sight-loss amongst the working age population in England and Wales [7]. Despite old age being a key determinant for blinding diseases, more than 400,000 people in the UK between the ages of 18 and 64 are estimated to experience some form of sight-loss [8]. Furthermore, almost 25,000 children in the UK under 14 years of age have conditions that cause visual problems or blindness [1], with Stargardt disease being the most prevalent form of juvenile macular dystrophy [9]. Stargardt disease is also untreatable.

The current cost of sight-impairment and blindness to the UK economy is estimated at £25 billion annually [2]. This reflects the growing number of individuals experiencing some form of visual problems and accounts for the direct costs of general ophthalmic services as well as the wider economic costs, including associated residential and community care, prescriptions, injurious falls and unemployment. Furthermore, only 1/4 partially sighted or blind individuals of working age are in employment [10], and despite disability legislation, exclusion from the workplace due to visual problems is estimated to cost £7.4 billion to the UK economy [2]. Ophthalmic services currently account for ~10% of all hospital outpatient appointments and more than 800,000 in-patient procedures each year, serving as a major contributor towards the £3.9 billion cost to the NHS [1, 2]. Currently, there are ~1 million anti-VEGF injections performed in NHS England per year, which is estimated to cost the NHS more than £500 million annually [11].

The UK model for eye research constitutes funding from independent organisations including UK Research and Innovation (UKRI) and eye charities. In a project funded through the UKRI QR Strategic Priorities Fund via Public Policy Southampton, we delved further into the mechanisms that support eye research in the UK. Using data from the 2018 UK Health Research Analysis online tool [12], we discovered that of the £32.6 million invested in eye research in 2018, 1/3 was provided through a mixture of eye research charities alongside larger charitable organisations such as the Wellcome trust. The remaining 2/3 of funding was provided by UKRI, primarily via the MRC, the BBSRC and Innovate UK. However, in the context of the £2.56 billion targeted to health research, only 1.3% was specifically invested in eye research, which is equivalent to just 0.8% of the annual NHS cost and just 0.1% of the total UK economic cost of vision problems (Fig. 1a, b). Analysis of funding by eye charities revealed a surprising picture. The five largest UK eye charities provide critical support and advice to blind and partially sighted people, but do not currently fund any research into eye diseases. The combined average annual expenditure in the 2016–2020 period for these organisations approximate to £350 million per year. By contrast, comparative figures for the five largest UK eye research charities for the same period amounts to only £18 million (Fig. 2). To better understand this mismatch, a public survey conducted by Sight Research UK showed that ~1/3 of the participants erroneously believed that The Royal National Institute of Blind People, one of the largest UK eye charities, funded medical research. Furthermore, 16% of respondents thought that The Guide Dogs for the Blind Association (Guide Dogs), another large UK eye charity, also invested in eye research, highlighting a general lack of public awareness of how eye research is funded. To help address these issues, our study employed a professional film crew to create a short documentary to highlight the everyday realities for patients and the urgent need for better funding into eye research (MP4 file).

**Fig. 1:**
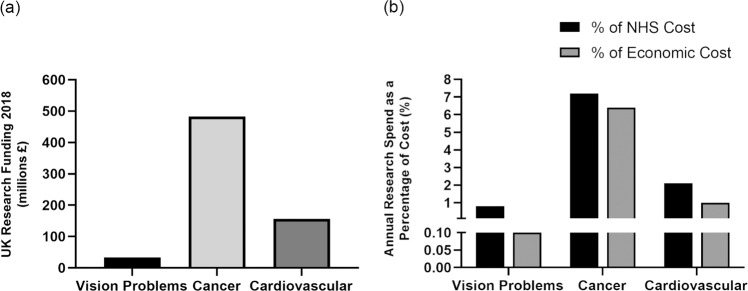
Research investment to address visual problems in the UK. **a** The comparatively low levels of research funding for visual problems vs. those for cancer [13] and cardiovascular diseases [14]. **b** The annual research spend for visual problems compared to those for cancer and cardiovascular diseases as a percentage of their National Health Service (NHS) and UK economic cost.

**Fig. 2:**
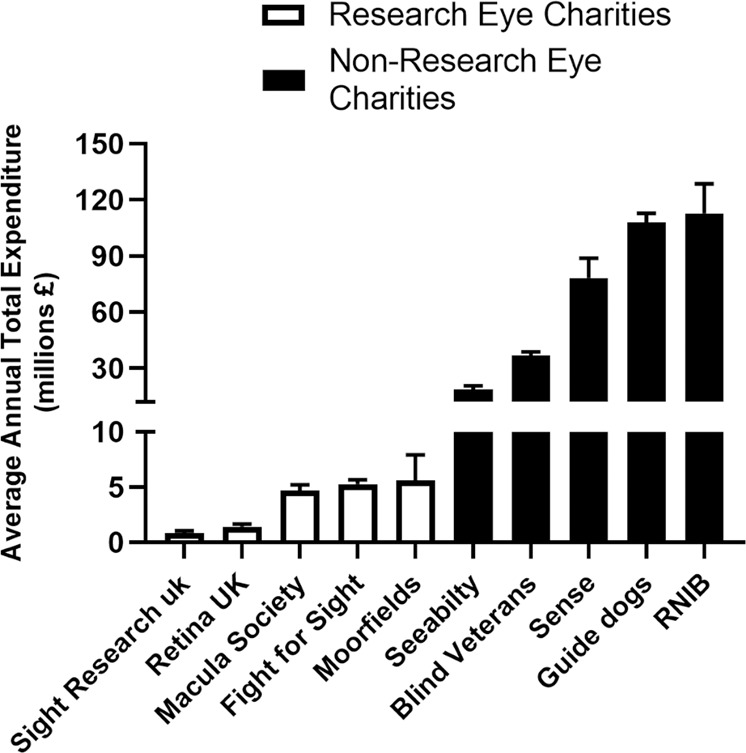
The average annual total expenditure by UK eye charities between 2016 and 2020. The combined average annual expenditure of the five largest UK eye charities consisting of SeeAbility, Blind Veterans UK, Sense, the Guide Dogs for the Blind Association and the Royal National Institute of Blind People (RNIB), approximate to £350 million per year. By contrast, the equivalent figures for the largest five UK eye research charities, which includes Sight Research UK, Retina UK, the Macular Society, Fight for Sight and the Moorfields Eye Charity, amount to only £18 million.

Despite recent advances in identifying the genetic basis of eye disease and insights into their molecular and cellular aetiologies, the development of meaningful new treatments will be slower to emerge without increased investment. If AMD prevalence could be reduced by 1% each year by 2050, health related costs would be reduced by £1.2 billion [2]. The limited investment for eye research in the UK is fragmented, and lacks the substantial and sustained government funding and overview that is provided by the National Eye Institute in the United States. A public misunderstanding of eye research funding in the UK is also a likely contributor to its under-investment. The increasing unmet need of patients as well as the wider societal and economic costs of visual defects and blindness are unlikely to be addressed without an urgent attempt to surmount these fundamental issues.

## Supplementary information

Supplemental Video

Impact of sight-loss and lack of funding for eye research in the UK
