# Supplementary material for: The disparity between funding for eye research vs. the high cost of sight-loss in the UK
Source: Eye (Lond). 2022 Sep 27;37(4):584–6. doi: 10.1038/s41433-022-02228-7 (PMC9998460; doi:10.1038/s41433-022-02228-7)
Supplement: Supplementary file 1 — Supplemental Video [file 41433_2022_2228_MOESM1_ESM.docx]

**Supplemental Video**

**Impact of sight-loss and lack of funding for eye research in the UK.**

A short documentary to highlight the everyday realities for patients with blinding diseases and the urgent need for better funding into eye research.
